# Smartphone usage behaviors and their association with De Quervain’s Tenosynovitis (DQT)among college students: a cross-sectional study in Guangxi, China

**DOI:** 10.1186/s12889-023-16808-z

**Published:** 2023-11-16

**Authors:** Xinyu Nie, Lihong Huang, Jun Hou, Anyuan Dai, Lihuan He, Puxiang Zheng, Zhimao Ye, Shiming Zhang, Guangqi Zhou, Jun Zhang, Qikai Hua

**Affiliations:** 1 Department of Spine Surgery, The second hospital of Jilin University, Changchun, China; 2 Department of Bone and Joint Surgery, The First Affiliated Hospital of Guangxi Medical University, Nanning, China; 3 Guangxi Diabetic Foot Salvage Engineering Research Center, The First Affiliated Hospital of Guangxi Medical University, Nanning, China; 4 Stomatology College of Guangxi Medical University, Nanning, China; 5 Department of Orthopedics, Sinopharm Dongfeng General Hospital of Hubei University of Medicine, Shiyan, China; 6 Xianning Medical College, National Demonstration Center for Experimental General Medicine Education, Hubei University of Science and Technology, Xianning, China

**Keywords:** Smartphone, Usage behaviors, De Quervain’s Tenosynovitis, College Students

## Abstract

**Background:**

The growing prevalence of smartphone use among college students in China has led to health concerns, including De Quervain’s Tenosynovitis (DQT). However, the specific smartphone usage behaviors contributing to DQT remain poorly understood. This study aimed to explore the relationship between smartphone usage behaviors and DQT in college students.

**Methods:**

A cross-sectional study was conducted with 937 students from various majors in Guangxi between September 2021 and April 2022. Participants completed an online questionnaire assessing smartphone usage behaviors and their association with DQT. The Finkelstein test was employed to diagnose DQT.

**Results:**

Over half of the college students (52%) tested positive for DQT via Finkelstein’s test. Higher levels of smartphone usage time (6–8 h/day: OR = 4.454, 95%CI:1.662–12.229; ≥8 h/day: OR = 4.521, 95%CI:1.596–12.811), phone games (OR = 1.997, 95%CI:1.312–3.040), social media (OR = 2.263, 95%CI:1.795–3.833), and leisure activities (OR = 1.679, 95%CI:1.140–2.475) were significantly associated with an increased risk of DQT. Two specific gestures (Bilateral thumbs, BT: OR = 1.900, 95%CI:1.281–2.817; Bilateral thumbs-horizontal screen, BT-HS: OR = 1.872, 95%CI:1.244–2.818) and two screen sizes (5.0-5.5inch: OR = 2.064, 95%CI:1.108–3.846; 6.0-6.5inch: OR = 2.413, 95%CI:1.125–4.083) also exhibited a higher risk of DQT. Bilateral DQT was observed, with Gesture-BT identified as the primary risk factor.

**Conclusion:**

Our findings suggest that increased smartphone usage time, phone games, social media, and leisure activities elevate the risk of DQT among college students. Furthermore, two specific gestures and two screen sizes were also linked to a heightened DQT risk. To mitigate DQT development, college students should reduce smartphone usage time and adopt appropriate gestures.

## Introduction

Over the past decade, the proliferation of smartphones has transformed the way people communicate, learn, and entertain themselves, particularly among college students [1]. China, as the world’s largest smartphone market, has experienced a dramatic increase in smartphone users, with nearly every college student owning a smartphone by 2022 [2, 3]. Although smartphones have undeniably enhanced the lives of users through their diverse applications and features, this increased reliance on smartphones has raised concerns regarding addiction, mental health issues, and physical health problems such as De Quervain’s tenosynovitis (DQT) [1, 4–7].

De Quervain’s tenosynovitis, initially identified by Fritz de Quervain in 1895, is a widespread tendinopathy affecting the upper extremity. [8, 9] This condition is characterized by thickening and myxoid degeneration of the tendon sheath in the first dorsal compartment of the wrist. Among young, active populations, the incidence rate is 1.0 case per 1,000 person-years for women and 0.6 cases per 1,000 person-years for men. In the general adult working population, its prevalence is 0.5% for men and 1.3% for women [8, 10, 11].

Numerous studies have attempted to explore the relationship between smartphone usage, including messaging, various applications, mobile gaming, and the development of DQT. However, many of these studies have failed to address crucial factors such as phone usage posture, screen size, and the influence of daily thumb/wrist activities or physical fitness [5–7, 12]. Furthermore, the rapid evolution of smartphones and their associated applications results in changing usage patterns across different populations, necessitating the consideration of both temporal and cultural differences when analyzing this relationship [13].

This study aims to provide an updated and comprehensive analysis of the association between smartphone usage behaviors and DQT among Chinese college students. By incorporating previously overlooked factors and acknowledging the dynamic landscape of smartphone technology and applications, we seek to deepen our understanding of the potential risks associated with excessive smartphone use. Our goal is to offer evidence-based recommendations that may help mitigate the development of DQT in this population, ultimately fostering healthier smartphone habits for college students.

## Methods

### Participants and Study Design

We conducted an anonymous online survey using Wen Juan Xing, a platform akin to Qualtrics, SurveyMonkey, or Cloud Research, which provides online questionnaire design and survey services for various entities. The survey took place from September 2021 to April 2022, targeting 2000 college students at Guangxi Medical University. Wen Juan Xing’s use in previous academic research attests to its credibility and effectiveness as a data collection tool [14, 15]. The Ethics Committee in Research at Guangxi Medical University approved this research project(2023-E173-01). This study obtained the informed consent of all participants.

### Measures

The study questionnaire was developed based on a thorough review of existing literature and the current circumstances of college students. A pilot study with 30 non-medical students was conducted offline to evaluate their comprehension of the questions, average completion time, question relevance, and the feasibility of learning the Finkelstein maneuver independently. The pilot study results guided appropriate modifications before launching the web-based questionnaire, which consisted of four parts:

(1) Daily life aspects, such as thumb/wrist physical training in the past 14 days, previous diagnoses of thumb/wrist diseases, and time spent using objects like mice, keyboards, and micropipettes that involve thumb/wrist activity.

(2) Smartphone usage behaviors, including time spent using the phone (excluding long video watching), primary phone usage, screen size, and phone usage gestures. Students could select 1–2 options for usage and gestures.

(3) Wrist/thumb health (for both dominant and non-dominant hands), encompassing symptoms in the past 14 days, symptom types, Finkelstein test results, and Visual Analog Scale (VAS) scores for the Finkelstein test.

(4) Using web-based questionnaire platforms, a GIF image demonstrated the proper execution of the Finkelstein test [10, 16, 17], enabling participants to evaluate their abductor pollicis longus and extensor pollicis brevis tendons for signs of tenosynovitis and allowing a more accurate self-assessment of their wrist/thumb health.

### Exclusion criteria

Participants meeting the following criteria were excluded from the study:

1. Thumb/wrist subjected to heavy physical training in the past 14 days (184 options).
2. Previous diagnosis of thumb/wrist disease (98 options).
3. Thumb/wrist usage of other objects, such as mice, keyboards, micropipettes, etc., exceeding 4 h per day (307 options).

### Data collection

The study employed entirely electronic methods, using web-based questionnaire platforms. Participants completed the questionnaire via their smartphones, with data collected on the website immediately after submission.

### Statistical analyses

Data were collected, organized, coded, and entered into a master sheet on a personal computer. All statistical analyses were performed using SPSS version 20.0 (IBM Corp, Chicago, IL, USA). Continuous variables were compared using the independent-sample *t* test. Categorical data were analyzed by the chi-square test or continuous correction chi-square test. Variables were tested for significance between groups, with a p-value of < 0.05 considered statistically significant. Logistic regression analysis was used to determine absolute risk factors and adjust for age and gender.

## Results

A total of 1,438(73.9%) students initially responded to the study. After applying the exclusion criteria, 937 students remained for data analysis. The participants were from different majors at Guangxi Medical University, with 404 (43.1%) males and 533 (56.9%) females. The age difference between males (20.60 ± 1.98 years) and females (20.69 ± 2.175 years) was not significant (p = 0.212).

### Differences between the positive and negative groups

Of the 937 students, 488 had positive Finkelstein’s test results, yielding a positivity rate of 52.1%. No statistically significant differences were observed between students with positive and negative Finkelstein’s test results concerning gender (p = 0.077) and age (p = 0.212) (Table 1).

**Table 1:** Investigation results of Finkelstein’s test

	Positive(n = 488)		Negative(n = 449)		P-value
	n	%	n	%	
**Gender**					
Male	197	40.4	207	46.1	0.077
Female	291	59.6	242	53.9	
**Age**					
< 18 years old	10	2	10	2.2	0.212
18<-≤20 years old	247	50.6	231	51.4	
21<-≤24 years old	199	40.8	162	36.1	
24<-≤26 years old	28	5.7	37	8.2	
>26 years old	4	0.8	9	2.0	
**Usage Times**					
< 2 h/day	6	1.2	20	4.4	**< 0.001**
2<-≤4 h/day	37	7.5	105	23.2	
4<-≤6 h/day	146	29.7	169	37.4	
6<-≤8 h/day	192	39.1	105	23.2	
>8 h/day	107	21.8	40	11.0	
Average	6.46 ± 1.86		5.13 ± 2.02 h		
**Uses**					
Phone Games	163	16.4	54	6.4	**< 0.001**
Social Media^1^	391	39.4	299	35.3	
Leisure activities^2^	322	32.5	310	36.6	
Study	116	11.7	183	21.6	
**Gesture** ^**3**^					
UTDP	266	37.6	255	38.9	**< 0.001**
UT-OA	63	8.9	83	12.7	
BT	232	32.8	150	22.9	
BT-HS	86	12.2	76	11.6	
UI-OA	60	8.5	92	14.0	
**Screen Size** (inch)					
<5.0	22	4.5	42	9.3	**0.02**
5.0<-≤5.5	186	38.1	149	33.2	
5.5<-≤6.0	109	22.3	126	27.9	
6.0<-≤6.5	129	26.4	92	20.4	
6.5<-≤7.0	40	8.2	34		
>7.0	2	0.5	6	1.3	
**Symptoms** ^**4**^					
Yes	82	16.8	49	10.9	**0.009**
No	406	83.2	400	80.1	

**Fig. 1:**
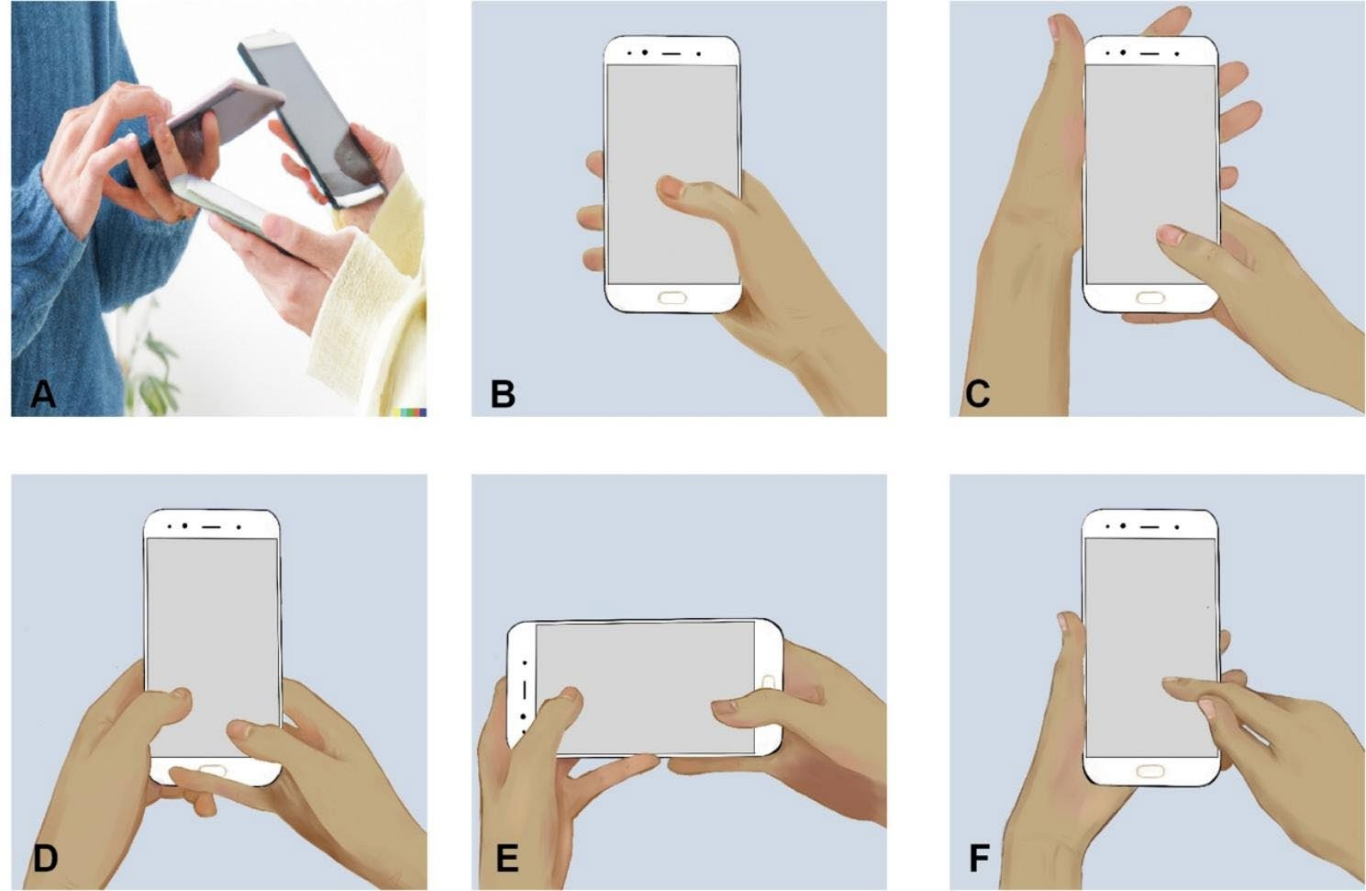
Figure of smartphone usage gestures. This figure shows different common postures of using a cell phone. Figure 1-**A** is a schematic diagram of the use of a smartphone. Figure 1-**B**: Unilateral Thumb-Dependent Posture, UTDP; Fig. 1-**C**: Unilateral Thumb- Opposite hand accompaniment, UT-OA; Fig. 1-**D**: Bilateral thumbs, BT; Fig. 1-**E**: Bilateral thumbs (horizontal screen), BT-HS;Figure-1:**F**-Unilateral index finger– Opposite hand accompaniment, UI-OA.

Notes:

1. Includes WeChat, QQ, Weibo, Message, Voice call et al.

2. Includes watching videos, reading eBooks, listening music, going shopping, et al.

3. Gestures are plotted in Fig. 1.

4. Symptoms: Discomfort (sore, stiff, snap, pain, et al.) in the thumb or wrist in the past 14 days

A total of 26, 142, 315, 297, and 147 students used their smartphones for ≤ 2 h/day, 2-4 h/day, 4-6 h/day, 6-8 h/day, and ≥ 8 h/day, respectively. Finkelstein’s test positivity rates significantly increased with smartphone usage time, from 23.1% for ≤ 2 h/day to 72.8% for ≥ 8 h/day (p < 0.01). Students with positive Finkelstein test results had a mean usage time of 6.46 ± 1.86 h, significantly longer than that of the negative students (5.13 ± 2.02 h, p < 0.001) (Table 1).

Regarding smartphone usage purposes, statistically significant differences were observed between the positive and negative groups (p < 0.001). In the positive group, the most common purposes were phone games (16.4%), social media (39.4%), leisure activities (32.5%), and study (11.7%). In the negative group, the distribution was phone games (6.4%), social media (35.5%), leisure activities (36.6%), and study (21.6%) (Table 1).

Statistically significant differences were also found in smartphone usage gestures between the positive and negative groups (p < 0.001). In the positive group, the most common gestures were Gesture-UTDP (37.6%), Gesture-UT-OA (8.9%), Gesture-BT (32.8%), Gesture-BT-HS (12.2%), and Gesture-UI-OA (8.5%). In the negative group, the distribution was Gesture-UTDP (38.9%), Gesture-UT-OA (12.7%), Gesture-BT (22.9%), Gesture-BT-HS (11.6%), and Gesture-UI-OA (14.0%).

A total of 64, 335, 235, 321, 74, and 8 students used smartphones with screen sizes ≤ 5.0 inches, 5-5.5 inches, 5.5-6 inches, 6-6.5 inches, 6.5-7 inches, and ≥ 7.0 inches, respectively. Finkelstein’s test positivity rates were 34.4%, 55.5%, 46.4%, 58.4%, 54.1%, and 25%, respectively, and were statistically different between the positive and negative groups (p = 0.02).

Furthermore, a significantly higher number of students in the positive group (82 or 16.8%) experienced symptoms before the test in the past 14 days compared to the negative group (49 or 10.9%, p = 0.009) (Table 1).

### Associations between smartphone usage behaviors and DQT

Logistic regression analysis of the factors (usage time, purpose of smartphone, gestures, screen size, and symptoms) (Table 2)revealed that usage time (6-8 h/day, p = 0.004, OR = 4.454, 95% CI: 1.662–12.229; ≥8 h/day, p = 0.004, OR = 4.524, 95% CI: 1.596–12.811), the purpose of the smartphone (phone games, p = 0.001, OR = 1.997, 95% CI: 1.312–3.040; social media, p < 0.001, OR = 2.263, 95% CI: 1.795–3.833; leisure activities, p = 0.009, OR = 1.679, 95% CI: 1.140–2.475), gestures (Gesture-BT, p = 0.001, OR = 1.90, 95% CI: 1.281–2.817; Gesture-BT-HS, p = 0.003, OR = 1.872, 95% CI: 1.244–2.818), and screen size (5-5.5 inches, p = 0.022, OR = 2.064, 95% CI: 1.018–3.846; 6-6.5 inches, p = 0.021, OR = 2.413, 95% CI: 1.125–4.083) were statistically significantly associated with increased DQT.

**Table 2:** Association between gender, ages, uses gestures, screen size activity and symptoms risk of DQT (n = 937)

	N	OR	High risk of DQT	P-value			
			95%CI				
**Usage Times**							
< 2 h/day	6	Ref					
2<-≤4 h/day	37	0.937	0.326–2.697	0.904			
4<-≤6 h/day	146	2.003	0.732–5.480	0.176			
6<-≤8 h/day	192	4.454	1.662–12.229	**0.004**			
>8 h/day	107	4.521	1.596–12.811	**0.004**			
**Uses**							
Phone Games	163	1.997	1.312–3.040	**0.001**			
Social Media	391	2.263	1.795–3.833	**< 0.001**			
Leisure Activities	322	1.679	1.140–2.475	**0.009**			
Study	116						
**Gestures**							
UTDP	266	1.156	0.786–1.702	0.461			
UT-OA	63	0.892	0.559–1.422	0.630			
BT	232	1.900	1.281–2.817	**0.001**			
BT-HS	86	1.872	1.244–2.818	**0.003**			
UI-OA	60	0.835	0.536–1.299	0.423			
**Screen Size** (inch)							
<5.0	22	Ref					
5.0<-≤5.5	186	2.064	1.108–3.846	**0.022**			
5.5<-≤6.0	109	1.591	0.840–3.016	0.154			
6.0<-≤6.5	129	2.413	1.125–4.083	**0.021**			
6.5<-≤7.0	40	1.725	0.810–3.673	0.158			
>7.0	2	0.766	0.126–4.653	0.773			
**Symptoms**	82	1.387	0.905–2.126	0.133			

Notes:

1. Models were adjusted for gender, age.

2. Bold values indicate p < 0. 05.

### Differences and associations between unilateral and bilateral DQT

Among the students with positive Finkelstein’s test results, 42.21% exhibited bilateral DQT. In terms of gender, 119 (52.7%) bilateral DQT students were male, compared to unilateral DQT students, with a statistically significant difference (p = 0.01). Among the unilateral positive group, 54.4% were aged 18–20 years, compared to 46.5% in the bilateral group (p = 0.026). Gesture-BT (33.6%) and Gesture-BT-HS (23.1%) were more commonly used by the bilateral DQT group than the unilateral group (Gesture-BT: 26.7%, Gesture-BT-HS: 15.0%), with a statistically significant difference (p = 0.04) (Table 3).

**Table 3:** Investigation results of Unilateral and Bilateral DQT

	Unilateral		Bilateral		P-value
	N	%	N	%	
**Gender**					
Male	89	34.1	119	52.7	0.01
Female	172	65.9	108	47.3	
**Age**					
< 18 years old	3	1.1	7	3.1	**0.026**
18<-≤20 years old	142	54.4	105	46.5	
21<-≤24 years old	97	37.2	102	45.1	
24<-≤26 years old	20	7.3	8	3.5	
>26 years old	1		3	1.8	
**Usage Times**					
< 2 h/day	3	1.1	3	1.3	0.143
2<-≤4 h/day	13	5.0	24	10.6	
4<-≤6 h/day	76	28.7	70	31.0	
6<-≤8 h/day	109	41.8	83	36.7	
>8 h/day	41	23.4	46	20.4	
**Uses**					
Phone Games	66	13.3	81	17.8	0.105
Social Media	216	43.6	174	38.2	
Leisure Activities	150	30.3	151	33.2	
Study	63	12.7	49	10.8	
**Gesture**					
UTDP	161	41.4	104	28.0	**0.04**
UT-OA	35	8.9	28	7.5	
BT	104	26.7	125	33.6	
BT-HS	58	15.0	86	23.1	
UI-OA	31	8.0	29	7.8	
**Screen Size** (inch)					
<5.0	15	5.7	7	3.1	0.09
5.0<-≤5.5	100	38.3	86	38.1	
5.5<-≤6.0	67	25.7	42	18.6	
6.0<-≤6.5	57	21.8	72	31.4	
6.5<-≤7.0	21	8.0	19	8.4	
>7.0	1	0.04	1	0.4	
**Symptoms**					
Yes	41	84.3	41	81.9	0.474
No	220	15.7	186	18.1	

Logistic regression analysis incorporating factors (gender, age, usage time, purpose of smartphone, gestures, screen size, and symptoms) (Table 4) revealed that Gesture-BT (p = 0.002, OR = 2.221, 95% CI: 1.324–3.375) was statistically significantly associated with increased bilateral DQT.

**Table 4:** Association between gender, age, gestures, screen size activity, and symptoms risk of Bilateral DQT (n = 488)

	N	OR	High risk of DQT	P-value
			95%CI	
**Gender**		1.575	1.048–2.366	**0.029**
**Usage Times**				
< 2 h/day	3	Ref		
2<-≤4 h/day	13	2.022	0.357–11.438	0.426
4<-≤6 h/day	76	2.204	0.955–5.085	0.064
6<-≤8 h/day	109	1.235	0.714–2.133	0.450
>8 h/day	41	1.029	0.619–1.712	0.911
**Uses**				
Phone Games	66	1.395	0.768–2.531	0.274
Social Media	216	0.739	0.416–1.314	0.303
Entertainment- Activities	150	1.335	0.782–2.278	0.289
**Gestures**	63			
UTDP	161	0.704	0.417–1.186	0.187
UT-OA	35	0.982	0.494–1.951	0.959
**BT**	104	**2.221**	**1.324–3.375**	**0.002**
BT-HS	58	1.632	0.978–2.271	0.061
UI-OA	31	1.518	0.776–2.969	0.223
**Screen Size** (inch)				
<5.0	15	Ref		
5.0<-≤5.5	100	0.548	0.028–10.853	0.693
5.5<-≤6.0	67	0.952	0.055–16.425	0.973
6.0<-≤6.5	57	0.720	0.041–12.567	0.822
6.5<-≤7.0	21	1.368	0.079–23.705	0.830
>7.0	1	0.892	0.049–16.216	0.938
**Symptoms**	41	1.306	0.620–1.731	0.894

Notes:

1. Models were adjusted for age.

2. Bold values indicate p < 0.05.

## Discussion

De Quervain’s Tenosynovitis (DQT) is a common condition caused by repetitive motion of the thumb, leading to microdamage of collagen fibers and eventually triggering a repair reaction [10]. DQT is anatomically influenced by dorsiflexion and ulnar deviation of the wrist and is associated with factors such as time, use, and occupation [18, 19]. Among college students, with the rise of prolonged use of smart devices like smartphones, computers, iPads, and hand movement-related activities such as ping pong, playing musical instruments like the guitar, writing, painting, and hands-on experimental operations in scientific research, the prevalence of DQT has increased. In recent years, the usage of smartphones has become increasingly diverse, with younger populations being frequent users [5, 12]. However, the evolution of smartphone behaviors among college students, such as usage time, posture, screen size, and usage, requires an update of relevant studies.

Currently, there is no established standard test for diagnosing DQT. However, Finkelstein’s test is considered the most effective diagnostic tool for De Quervain’s Tenosynovitis at present. In the latest controlled study, the Finkelstein’s test with a specificity of 100% [17, 20]. The test involves holding the forearm in a neutral position and extending the wrist to the edge of the examiner’s table. The examiner then asks the subject to deviate their wrist, following which, the active ulnar side grasps the subject’s thumb and passively extends it into the palm. A positive Finkelstein’s test is indicated if the subject feels pain during the test. To ensure accurate data collection, we provided detailed Finkelstein’s test schematics in the questionnaire to enable each student to perform the test themselves.

In this study, we conducted an online survey to assess the prevalence of DQT among college students from various majors at Guangxi Medical University. To our knowledge, this study is the only one published that reports the different specific details.

association between usages and DQT. Our results revealed a high prevalence rate of 52.7%, which is slightly higher than the rates reported in previous studies conducted in China and Brazil [5, 12]. This finding could be attributed to the continuous evolution of smartphones and the latest research updates in the field.

### Gender, age, and time

For gender and age, this study showed no statistical difference, similar to the previous study. [5, 6] In the present study, we found a statistical difference in Finkelstein’s test results based on usage time. When the usage time is 6-8 h/day, smartphone use presents a higher risk of DQT (OR = 4.454); the more time (≥ 8 h/day), the higher the risk (OR = 4.521). As our usage time is a combination of phone usage time, it is different in the data from just playing the game. However, there is no doubt that usage time is one of the important factors that cause DQT.

### Phone uses

The dramatic shift in smartphone usage necessitates a more comprehensive approach to examining the links between social or gaming activities and disease, as single associations are no longer sufficient to prompt a reevaluation of the extent of smartphone use [21]. In our study, we found that the risk of developing DQT varies depending on the type of smartphone use. Prior research has increasingly focused on the effects of mobile phone software on individuals. However, the China Internet Network Information Center (CNNIC) has reported that the number of applications in China is expected to reach 3.02 million by 2022(http://cnnic.com.cn/). studying the impact of a specific app may not be sufficiently objective or convenient for research purposes, as a result, we classified cell phone use into four categories: common mobile games, leisure activities including popular social software, daily life activities, and professional work (Table 1). Our results showed that the Finkelstein test positive group differed significantly from the negative group in terms of cell phone use (< 0.001), with the positive group showing a preference for mobile games and the negative group spending more time on professional work. However, after a comprehensive analysis, we found that activities involving phone games, social media, and leisure had a higher risk of DQT, likely due to the repeated hand movements required for these activities. Furthermore, spending long hours (more than 6 h per day) on these activities can increase the risk of DQT, which is consistent with previous studies [5, 12].

### Gesture

The most direct anatomical factor affecting DQT is posture while using a mobile phone, yet few studies have investigated the relationship between changes in the incidence of DQT and changes in individual gestures. While previous researchers have referred to mobile phone gestures, there has been no specific study of the effect of this posture on DQT and how it relates to other factors such as screen size, time of day, etc. Figure 1 summarizes several common postures for holding a mobile phone that were included in our questionnaire and from which participants could choose. Each person will have 1–2 positions they prefer due to the different uses of the phone and personal habits, and the different gestures require a combination of strength between the thumb and wrist. Prolonged manipulation can lead to pathological changes. Kutsumi et al. studied the relationship between frictional resistance and wrist position in EPB and APL and found that the wrist obtained maximum sliding resistance in the maximum flexion or dorsiflexion position [22]. An increase in frictional resistance can easily lead to degenerative changes and mechanical injury, resulting in DQT [23, 24]. In past studies, only the postures that may be used for specific activities have generally been addressed, such as postures commonly used for mobile gaming, but no additional factors such as phone size, time of use, etc. have been linked [12]. In our study, there was a difference in the distribution of postures used by university students between the two groups (p < 0.001), and in our risk factor analysis, we found a higher risk of using both thumbs, mainly for mobile gaming and constant chatting. This prolonged and frequent use caused transitory thumb activity in a short time to trigger DQT. We recommend using the phone in a variety of positions and avoiding prolonged thumb manipulation to mitigate the risk of DQT.

### Screen size

In the early days of mobile phones, there was not a lot of research on screen size, as the non-smartphone era was all about buttons and no touchscreens. With the rapid development of smartphones in recent years, the size of mobile phone screens has gradually become larger. In previous studies, researchers have found that screen size has not been an influential factor in studies on the relationship between mobile gaming and DQT [12]. However, in our study, we found that a bias towards smaller 5-5.5 inches and a bias towards larger 6.0-6.5 inches had a higher risk, which may be related to the hand size of men versus women, however, additional research is required to substantiate this claim.

### Bilateral DQT and symptoms

This study is the first to examine the possibility of bilateral DQT in smartphone use, a phenomenon not previously reported in the literature, which highlights the growing impact of smartphone use on people’s health. In our frontal study, we found that up to 42.21% of the subjects in the positive group had positive DQT bilaterally, which is of concern and may be related to the prolonged use of both thumbs. However, this needs to be further investigated. In terms of risk factors for bilateral DQT, although our data show that only Gesture-BT is a risk factor, this may be related to the small amount of data we have. Theoretically, it is highly likely that prolonged double thumb chatting will also result in bilateral DQT.

In this study, we found that people in the positive group were more likely to have symptoms in the last 14 days, and appear bilaterally, which has not been reported before. This may mean that when you have discomfort in your thumb or wrist, you must stop using your phone and take a proper break.

There are several limitations to this study that warrant attention. (1) The study is implemented in medical University. The medical students might have more knowledge in health-related issues, which might cause sampling bias. (2) Although this study has excluded most of the major factors affecting the incidence of DQT among college students apart from mobile phone use, it cannot rule out the impact of other activities that last less than 4 h on the incidence of DQT. (3) Our analysis did not specifically account for the time of day or individual gestures during usage, which should be explored in future research. (4) The study participants used various mobile phone models with differing screen sizes, resolutions, and thicknesses, but only screen sizes were considered. (5) It is possible that different types of games may have varying impacts on DQT risk, as strategy games, for example, typically involve less thumb contact and movement (up and down). This factor was not considered prior to the investigation. Future studies should address these concerns and aim to corroborate our findings through a multicenter, large-sample prospective cohort study.

## Conclusion

This study highlights the significant prevalence of De Quervain’s Tenosynovitis (DQT) among college students, with a strong association to smartphone usage. Factors such as usage time, type of phone use, gesture, and screen size were found to contribute to the risk of developing DQT. The findings underscore the importance of raising awareness about DQT and implementing preventive measures, particularly for young adults who frequently use smartphones. As technology continues to advance, it is crucial to keep updating our understanding of its impact on health and well-being. Future research should address the limitations of the present study and provide a more comprehensive understanding of the risk factors associated with DQT in the context of smartphone use through multicenter, large sample prospective cohort studies.

## Data Availability

The datasets used during the current study are available from the corresponding author upon reasonable request.
